# Insomnia as a Symptom of Rapid Eye Movement-Related Obstructive Sleep Apnea

**DOI:** 10.3390/jcm9061821

**Published:** 2020-06-11

**Authors:** Tetsuro Hoshino, Ryujiro Sasanabe, Kenta Murotani, Reiko Hori, Mamiko Mano, Atsuhiko Nomura, Noriyuki Konishi, Masayo Baku, Aki Arita, Wojciech Kuczynski, Toshiaki Shiomi

**Affiliations:** 1 Department of Sleep Medicine and Sleep Disorder Center, Aichi Medical University Hospital, 1-1 Nagakute, Aichi 4801195, Japan; sasanabe@aichi-med-u.ac.jp (R.S.); rhori@aichi-med-u.ac.jp (R.H.); mano.mamiko.527@mail.aichi-med-u.ac.jp (M.M.); nomura.atsuhiko.689@mail.aichi-med-u.ac.jp (A.N.); konishi.noriyuki.011@mail.aichi-med-u.ac.jp (N.K.); mbaku@aichi-med-u.ac.jp (M.B.); arita.aki.014@mail.aichi-med-u.ac.jp (A.A.); toshiaki@aichi-med-u.ac.jp (T.S.); 2 Biostatistics Center, Graduate School of Medicine, Kurume University, 67 Asahimachi, Kurume, Fukuoka 8300011, Japan; kmurotani@med.kurume-u.ac.jp; 3 Department of Sleep Medicine and Metabolic Disorders, Medical University of Lodz, 92-216 Lodz, Poland; wojciech.kuczynski@umed.lodz.pl

**Keywords:** obstructive sleep apnea, rapid eye movement-related obstructive sleep apnea, Pittsburgh Sleep Quality Index, Epworth sleepiness scale

## Abstract

Rapid eye movement (REM)-related obstructive sleep apnea (OSA), a polysomnographic phenotype that affects 12–36% of OSA patients, is defined by apnea and hypopnea events that predominantly or exclusively occur during REM sleep. Recent studies indicated that REM-related OSA was associated with the development of nocturnal non-dipping of systolic and diastolic blood pressure, metabolic syndrome, diabetes, and depressive symptoms. However, to date, the association between REM-related OSA and insomnia still remains unclear. We investigated whether there was a difference between REM- and non-REM-related OSA in terms of insomnia-related sleep disturbance as measured by the Pittsburgh Sleep Quality Index (PSQI) in 1736 patients with OSA. REM-related OSA showed a significant association with increased PSQI in all adjusted models. In the subgroup analysis, the coefficients of all models were higher in female than in male patients with REM-related OSA. Insomnia should be considered an important complaint in patients with REM-related OSA, and its indicators, such as the PSQI, should be included in routine diagnostic testing.

## 1. Introduction

Obstructive sleep apnea (OSA) is a common sleep disorder characterized by repetitive respiratory events, including apnea and hypopnea, due to total or partial upper airway obstruction. Untreated OSA is associated with various comorbidities, daytime sleepiness, and increased mortality [1].

Rapid eye movement (REM)-related OSA, a polysomnographic phenotype that affects 12–36% of OSA patients, is defined by apnea and hypopnea events that predominantly or exclusively occur during REM sleep [2,3]. The exact pathophysiology of REM-related OSA remains unclear to date, and its overall severity as defined by the apnea–hypopnea index (AHI) tends to be mild to moderate. Clinically, it should be distinguished from non (N)-REM-related OSA because recent studies have indicated that REM-related OSA is associated with the development of nocturnal non-dipping of systolic and diastolic blood pressure, metabolic syndrome, and diabetes [4,5]. Moreover, other studies have indicated that REM-related OSA is more common in females, particularly those presenting with depressive symptoms [6,7].

The association between insomnia and OSA was first described by Guilleminault et al. in 1973 [8]. Insomnia, which has a reciprocal relationship with depression, has a prevalence of 39–58% in patients with OSA [9]. Therefore, we formulated the hypothesis that insomnia may be one of the chief complaints of REM-related OSA patients. However, to date, the association between REM-related OSA and insomnia is unclear. Consequently, in this study, we investigated whether there was a difference between REM-related and NREM-related OSA in terms of insomnia-related sleep □ disturbance as measured by the Pittsburgh Sleep Quality Index (PSQI), which is a valid and effective instrument in the assessment of insomnia in various populations [10,11,12].

## 2. Materials and Methods

### 2.1. Ethical Approval

This study was conducted in accordance with the amended Declaration of Helsinki. All procedures were performed in accordance with the ethical standards of the institutional review board (IRB) of Aichi Medical University Hospital, which approved the protocol of the study (approval number 2019-183).

The IRB granted a waiver of patients’ informed consent because of the non-invasive and retrospective nature of the study. Nevertheless, we published an outline of the study plan for public viewing on the Aichi Medical University website to provide patients with the opportunity to object to the use of their data. However, none of the patients objected to the use of their data in the study.

### 2.2. Study Population

**Figure 1:**
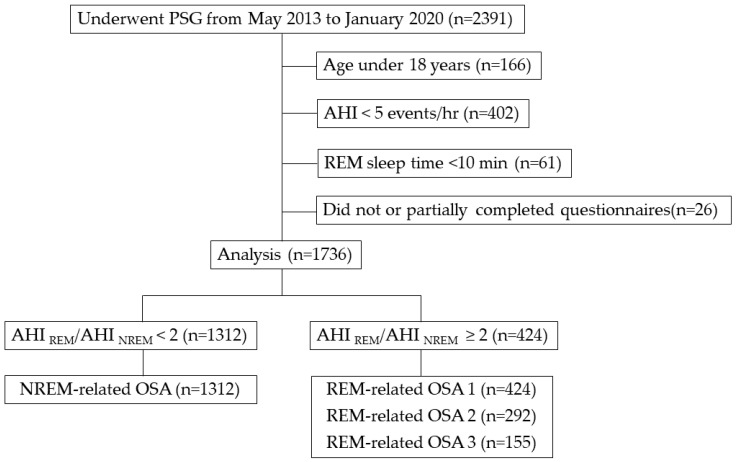
Flowchart of patients who underwent polysomnography (*n* = 2391) in this study. PSG, polysomnography; AHI, apnea–hypopnea index; REM, rapid eye movement; NREM, non-rapid eye movement; OSA, obstructive sleep apnea; REM-related OSA 1: overall AHI ≥ 5 and AHI during REM (AHI_REM_)/AHI during NREM (AHI_NREM_) ≥ 2; REM-related OSA 2: overall AHI ≥ 5, AHI_REM_/AHI_NREM_ ≥ 2, and AHI_NREM_ < 15; and REM-related OSA 3: overall AHI ≥ 5, AHI_REM_/AHI_NREM_ ≥ 2, AHI_NREM_ < 8, and REM sleep duration >10.5 min.

A total of 2391 patients with suspected sleep-related breathing disorders who underwent polysomnography at the Department of Sleep Medicine and Sleep Disorders Center of the Aichi Medical University Hospital in Aichi, Japan, from May 2013 to January 2020 were included in this study. The exclusion criteria were as follows: (1) patients <18 years of age; (2) patients with AHI < 5; (3) patients with a REM sleep time of less than 10 min; and (4) patients who did not or partially completed our sleep-related questionnaires. Based on these criteria, we excluded 166, 402, 61, and 26 patients, respectively. This resulted in 1736 adult OSA patients who were finally enrolled in the study (Figure 1).

### 2.3. Data Collection

Nocturnal polysomnography was performed with the Alice 5 or 6 System (Respironics Inc., Murrysville, PA, USA), PSG-1100 (Nihon Kohden Co., Tokyo, Japan) and SOMNOscreen plus PSG system (Somnomedics., Randersacker, Germany). The following biological variables were continuously monitored: electrocardiogram, electroencephalogram, chin and anterior tibialis electromyogram, bilateral electrooculogram, airflow measured with a nasal thermistor and prong pressure, respiratory effort measured on the basis of thoracic and abdominal movements, body position, snoring sound, and arterial oxygen saturation. Respiratory events, including apnea and hypopnea, and other polysomnographic parameters were scored manually by specialized sleep technologists, based on the American Academy of Sleep Medicine Manual for the Scoring of Sleep and Associated Events: Rules, Terminology, and Technical Specifications, version 2.0 [13]. OSA was defined as AHI ≥ 5 according to the International Classification of Sleep Disorders (second edition) criteria [14]. In reference to previous reports, we used three different definitions to determine REM-related OSA, according to progressively more stringent defining criteria:

- REM-related OSA 1: overall AHI ≥ 5 and AHI during REM (AHI_REM_)/AHI during NREM (AHI_NREM_) ≥ 2;
- REM-related OSA 2: overall AHI ≥ 5, AHI_REM_/AHI_NREM_ ≥ 2, and AHI_NREM_ < 15;
- REM-related OSA 3: overall AHI ≥ 5, AHI_REM_/AHI_NREM_ ≥ 2, AHI_NREM_ < 8, and REM sleep duration >10.5 min [3,15,16,17].

Patients with AHI_REM_/AHI_NREM_ < 2 were defined as NREM-related OSA.

### 2.4. Questionnaires

At their first visit to our department, patients were routinely asked to complete the Epworth Sleepiness Scale (ESS) and PSQI questionnaires. The ESS questionnaire consists of eight self-rated items, each to be scored from 0 to 3, which measure a patient’s likelihood of daytime sleepiness in various commonly encountered situations. No specific time frame was defined to complete the questionnaires. The resulting ESS is the sum of the scores of these individual items and ranges from 0 to 24 [18]. The PSQI is a 19-item self-rated questionnaire for evaluating insomnia-related sleep disturbance over the previous month. The 19 items are grouped into seven components, i.e., sleep duration, sleep disturbance, sleep latency, daytime dysfunction due to sleepiness, sleep efficiency, overall sleep quality, and sleep medication use. Each of these components’ scores are added up to obtain the final score that ranges from 0 to 21 [10].

### 2.5. Statistical Analysis

Continuous variables are expressed as the median and interquartile range (25th percentiles–75th percentiles). The comparisons between REM-related OSA and NREM-related OSA in terms of patient characteristics and polysomnography parameters were conducted using the Mann–Whitney U test. The Chi-square test was used for categorical variables.

Multiple linear regression analysis was used to examine potential associations between REM-related OSA and PSQI [19]. The dependent variable was the presence or absence of REM-related OSA, and the independent variable was the PSQI. Each model was adjusted for covariates which were selected based on their established clinical difference between individuals with REM-related OSA and NREM-related OSA [2,3,20]. In Model 1, age, gender, and BMI were selected as covariates; in Model 2-a, AHI_REM_ was also included as a covariate, alongside the covariates that were selected in Model 1; in Model 2-b, AHI_NREM_ was also included as a covariate, alongside the covariates that were selected in Model 1.

Furthermore, a subgroup analysis by gender was performed. For this analysis, the dependent variable was the presence or absence of REM-related OSA, and the independent variable was the PSQI. The covariates were selected based on their established clinical relationship with gender differences in OSA [3]. In Model 1, age and body mass index (BMI) were selected as covariates; in Model 2-a, AHI_REM_ was also included as a covariate, alongside the covariates that were selected in Model 1; in Model 2-b, AHI_NREM_ was also included as a covariate, alongside the covariates that were selected in Model 1. All analyses were performed separately for each definition of REM-related OSA.

Coefficients are presented with their corresponding 95% confidence interval. All comparisons were two-tailed, and a *p* value < 0.05 was considered statistically significant.

Statistical analyses were performed using SAS 9.4 (SAS Institute Inc., Cary, NC, USA).

## 3. Results

Table 1 shows the comparison between the clinical characteristics and polysomnographic findings in patients with REM-related OSA 1–3 and those in patients with NREM-related OSA. The proportion of female patients among REM-related OSA 1, 2, and 3 was significantly higher (41.0%, 42.5%, and 49.0%, respectively) than that among patients with NREM-related OSA (19.2%). Regarding the polysomnographic findings, compared to patients with NREM-related OSA, patients with REM-related OSA 1, 2, and 3 showed significantly lower AHI, AHI_NREM_, cumulative percentage of time spent at an oxygen saturation below 90%, and arousal index. Among the questionnaires, the PSQI showed significantly higher scores for all patients with REM-related OSA 1, 2, and 3 than for patients with NREM-related OSA. The same finding was true for females when the analysis was performed according to gender. With respect to male patients, those with REM-related OSA 2 and 3 had significantly higher PSQI scores than those with NREM-related OSA.

**Figure 2:**
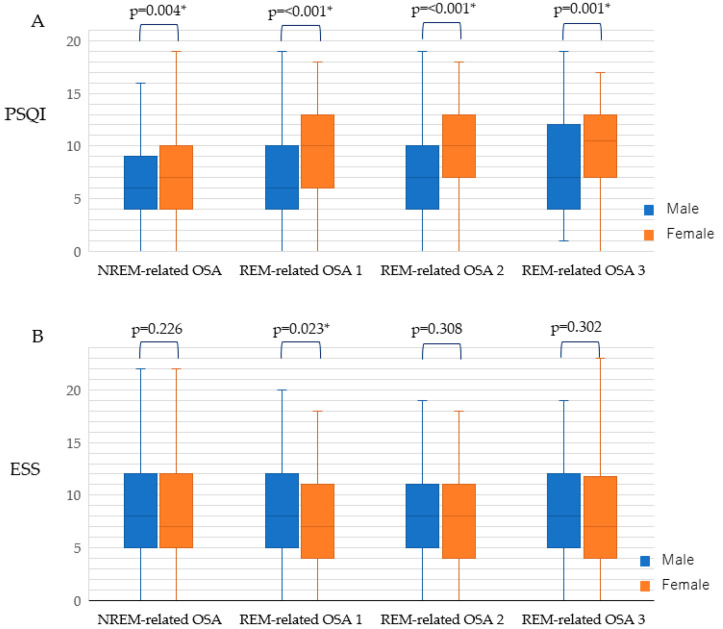
Gender difference in relation to (**A**) PSQI and (**B**) ESS for NREM-related OSA and REM-related OSA 1, 2, and 3. Date are shown as box plots (the central line of the box represents the median; the lower and upper limits of the box represent the 25th and 75th percentiles, respectively, and the whiskers represent the minimum and maximum). PSQI, Pittsburgh Sleep Quality Index; ESS, Epworth Sleepiness Scale; * *p* < 0.05.

Gender differences in ESS and PSQI for NREM-related OSA and REM-related OSA 1, 2, and 3 are shown in Figure 2. Female patients with NREM-related OSA and REM-related OSA 1, 2, and 3 had significantly higher PSQI than did the corresponding male patients. With respect to male patients, those with REM-related OSA 1 had significantly higher ESS scores than the corresponding female patients.

Table 2 shows the results of the multiple linear regression analysis for the association between PSQI and REM-related OSA 1, 2, and 3. The presence of REM-related OSA 1, 2, and 3 showed a significant association with increased PSQI, after adjusting for all covariates in the model.

Table 3 shows the results of the multiple linear regression analysis for the association between PSQI and REM-related OSA 1, 2, and 3 in male patients only. The presence of REM-related OSA 2 showed a significant association with increased PSQI, both in the unadjusted model (coefficient = 0.684, *p* = 0.032) and in the adjusted Model 1 (coefficient = 0.637, *p* = 0.045). The presence of REM-related OSA 3 showed a significant association with increased PSQI in the unadjusted model (coefficient = 1.057, *p* = 0.018) and adjusted Models 1 (coefficient = 1.048, *p* = 0.020) and 2-a (coefficient = 0.970, *p* = 0.031).

Table 4 shows the results of the multiple linear regression analysis for the association between PSQI and REM-related OSA 1, 2, and 3 in female patients only. The presence of REM-related OSA 1, 2, and 3 showed a significant association with increased PSQI for the unadjusted and all adjusted models. In the subgroup analysis by gender, the coefficients corresponding to REM-related OSA 1, 2, and 3 and each adjusted model were all higher in female patients than in male patients.

## 4. Discussion

In a previous study of 3234 OSA patients examined at our institution between 2004 and 2013, we found that the main symptom of REM-related OSA was not excessive daytime sleepiness [2]. Therefore, we added the PSQI as a recognized indicator of insomnia-related sleep disturbance in this study. To our knowledge, this is the first study that analyzed a potential association between REM-related OSA and PSQI.

The prevalence of REM-related OSA varies widely, ranging from 12% to 36% of all OSA cases, due to studies having inconsistent definitions that included various degrees of adjustment for AHI during NREM [2,3]. This makes it difficult to determine whether REM-related OSA is a distinct clinical entity. Therefore, we used three different definitions to classify REM-related OSA. The prevalence of REM-related OSA according to definitions 1, 2, and 3, based on progressively more stringent criteria, was 24.4%, 16.8%, and 8.9%, respectively.

We showed that REM-related OSA, especially in female patients, was associated with increased PSQI. Furthermore, the association was found to be stronger for REM-related OSA 3 than for REM-related OSA 1 and 2. Recent studies indicated that REM-related OSA is more frequently associated with depressive symptoms than NREM-related OSA [6,21,22]. Moreover, a previous cohort study demonstrated that the prevalence of depression was higher in female patients with REM-related OSA than in male patients [7]. At the same time, it was reported that 80% of patients with depression suffer from insomnia [23]. Although we found no direct association between REM-related OSA and insomnia, our results were consistent with these previous reports.

Recently, it was shown that OSA is caused by various combinations of the following four phenotypes: upper airway anatomical vulnerability, poor responsiveness of the upper airway dilator muscles to airway narrowing, instability in respiratory control, and a low arousal threshold [24]. Compared to during NREM sleep, there is an increased tendency for upper airway collapse during REM sleep, due to decreased muscle tone secondary to cholinergic inhibition of the hypoglossal nerve [25,26]. Therefore, reduced responsiveness and tone of the upper airway dilator muscles are regarded as key pathophysiological mechanisms in REM-related OSA [27,28]. On the other hand, Geckil and Ermis formulated the hypothesis that REM-related OSA may be a phenotype of classic OSA with a low arousal threshold [6]. Moreover, a previous study reported that insomnia likely results in a decreased arousal threshold [29]. Therefore, our results support this hypothesis. To develop precision medicine for OSA patients, further research is needed to elucidate the unclear pathophysiology of REM-related OSA.

Continuous positive airway pressure (CPAP) treatment is a symptomatic rather than radical treatment for OSA; therefore, maintaining adherence is crucial to obtain a beneficial effect. Although Su et al. indicated that general functional outcome showed greater improvements after CPAP therapy in patients with REM-related OSA, recent studies reported that adherence to CPAP therapy in patients with REM-related OSA was lower than that in patients with non-stage-specific OSA [16,17,30]. A previous study reported that insomnia among OSA patients reduced CPAP adherence [31]. No single factor which affects adherence to CPAP has been identified; however, the tendency for insomnia in patients with REM-related OSA might affect the low adherence to CPAP treatment.

In this study, there were no significant differences in the ESS between REM-related OSA 1–3 and NREM-related OSA. Previous studies have also reported no significant difference between the two groups in terms of daytime sleepiness as measured by the ESS [6,21,32].

Although one of the chief complaints of OSA patients that is indicated by the ESS is excessive daytime sleepiness, our results based on the PSQI identified insomnia rather than excessive daytime sleepiness as an important complaint in patients with REM-related OSA. Therefore, we recommend including insomnia indicators, such as the PSQI, as routine diagnostic tools for patients with REM-related OSA.

Our results should be interpreted within the limitations of our study. Firstly, a selection bias may have been present, since vocational, educational, and socio-economic levels which might affect insomnia symptoms were not assessed in this study. Secondly, a selection bias was also present because this study was conducted in a single center in Japan. Third, the effect of unknown confounding factors cannot be excluded because of the retrospective nature of the study. Moreover, we used only the PSQI for assessing insomnia-related sleep disturbance, although there are several other useful tools, such as the Insomnia Severity Index and Athens Insomnia Scale [33,34]. To validate our results, further studies evaluating insomnia using the Insomnia Severity Index or Athens Insomnia Scale are needed.

## 5. Conclusions

We found a significant relationship between REM-related OSA, especially when applying the strict definition of overall AHI ≥ 5, AHI_REM_/AHI_NREM_ ≥ 2, AHI_NREM_ < 8, and REM sleep duration >10.5 min, and increased PSQI. Among patients with REM-related OSA, females had a higher PSQI than males. We consider insomnia to be an important complaint in patients with REM-related OSA and recommend including insomnia indicators, such as the PSQI, in routine diagnostic testing of these patients.

## Figures and Tables

**Table 1:** Comparison of clinical characteristics and polysomnographic findings between patients with REM-related OSA and those with NREM-related OSA.

	(1) NREM-Related OSA (*n* = 1,312)	(2) REM-Related OSA 1 (*n* = 424)	(3) REM-Related OSA 2 (*n* = 292)	(4) REM-Related OSA 3 (*n* = 155)	*p* Value		
					(1) vs. (2)	(1) vs. (3)	(1) vs. (4)
Age	58 (48–70)	58 (46–70)	59 (46–70)	59 (45–71)	0.727	0.923	0.811
Female (%)	252 (19.2)	174 (41.0)	124 (42.5)	76 (49.0)	<0.001 *	<0.001 *	<0.001 *
BMI (kg/m^2^)	24.9 (22.5–28.3)	25.0 (22.3–28.6)	24.2 (21.9–27.5)	23.3 (21.5–26.8)	0.888	0.003 *	<0.001 *
TST (min)	460.5 (439.5–489.0)	456.0 (435.5–490.4)	459.0 (436.5–494.3)	463.0 (441.0–493.5)	0.149	0.560	0.823
Sleep latency (min)	6.5 (3.5–12.0)	7.5 (3.5–13.5)	7.5 (4.0–14.5)	7.5 (4.0–17.5)	0.032 *	0.021 *	0.006 *
Sleep efficiency (%)	80.3 (70.1–88.2)	83.5 (72.8–90.8)	84.1 (72.3–90.9)	82.0 (70.2–90.6)	<0.001 *	0.001 *	0.147
Stage REM (%)	15.9 (11.8–20.5)	17.0 (12.1–21.5)	17.6 (12.6–21.7)	17.6 (11.6–21.5)	0.025 *	0.005 *	0.074
Stage N1 (%)	46.4 (32.9–64.2)	28.4 (18.9–41.4)	24.9 (17.2–38.5)	23.0 (16.2–36.5)	<0.001 *	<0.001 *	<0.001 *
Stage N2 (%)	35.8 (20.0–48.0)	52.2 (40.8–61.8)	53.9 (41.7–63.4)	55.0 (44.9–64.2)	<0.001 *	<0.001 *	<0.001 *
Stage N3 (%)	0 (0–0.1)	0 (0–0.4)	0 (0–0.5)	0 (0–0.2)	0.064	0.053	0.363
AHI (events/h)	35.2 (18.7–55.6)	15.6 (9.1–22.6)	11.3 (7.8–15.9)	8.0 (6.8–9.6)	<0.001 *	<0.001 *	<0.001 *
AHI _REM_ (events/h)	34.3 (13.8–53.8)	37.9 (25.8–52.8)	30.0 (20.7–40.6)	23.7 (17.4–34.4)	<0.001 *	0.368	0.002 *
AHI _NREM_ (events/h)	35.0 (19.2–56.2)	10.8 (5.9–17.1)	7.4 (5.1–11.0)	5.2 (3.8–6.3)	<0.001 *	<0.001 *	<0.001 *
Lowest SpO_2_ (%)	82.0 (74.0–87.0)	84.0 (79.0–88.0)	86.0 (82.0–89.0)	88.0 (83.0–91.0)	<0.001 *	<0.001 *	<0.001 *
CT90 (%)	1.4 (0.1–7.1)	0.5 (0.1–2.0)	0.3 (0–0.9)	0.1 (0–0.5)	<0.001 *	<0.001 *	<0.001 *
Arousal Index (events/h)	38.2 (27.1–54.5)	23.3 (16.9–31.7)	20.9 (15.3–27.1)	18.8 (13.7–26.4)	<0.001 *	<0.001 *	<0.001 *
PLMI (events/h)	0 (0–7.7)	0 (0–11.0)	0 (0–12.4)	0 (0–10.8)	0.057	0.042 *	0.117
ESS	8 (5–12)	8 (5–11)	8 (5–11)	8 (5–12)	0.094	0.310	0.356
Male	8 (5–12)	8 (5–12)	8 (5–11)	8 (5–12)	0.782	0.723	0.903
Female	7 (5–12)	7 (4–11)	8 (4–11)	7 (4–11.8)	0.118	0.520	0.469
PSQI	6 (4–9)	7 (5–11)	8 (5–12)	9 (5–12)	<0.001 *	<0.001 *	<0.001 *
Male	6 (4–9)	6 (4–10)	7 (4–10)	7 (4–12)	0.089	0.044 *	0.035 *
Female	7 (4–10)	10 (6–13)	10 (7–13)	10.5 (7–13)	<0.001 *	<0.001 *	<0.001 *

Continuous variables are expressed as the median and interquartile range (25th percentiles–75th percentiles). Categorical variables are expressed as numbers (percentage); * *p* < 0.05. Diagnostic criteria: REM-related OSA 1: overall AHI ≥ 5 and AHI_REM_/AHI_NREM_ ≥ 2; REM-related OSA 2: overall AHI ≥ 5, AHI_REM_/AHI_NREM_ ≥ 2, and AHI_NREM_ < 15; and REM-related OSA 3: overall AHI ≥ 5, AHI_REM_/AHI_NREM_ ≥ 2, AHI_NREM_ < 8, and REM sleep duration > 10.5 min. Abbreviations: BMI, body mass index; CT90, cumulative percentage of time spent at oxygen saturation below 90%; PLMI, periodic limb movement index; SpO_2_, peripheral capillary oxygen saturation; TST, total sleep time.

**Table 2:** Association between three different definitions of REM-related OSA and the Pittsburgh Sleep Quality Index.

	REM-Related OSA 1			REM-Related OSA 2			REM-Related OSA 3		
	Coefficient	95% CI	*p* Value	Coefficient	95% CI	*p* Value	Coefficient	95% CI	*p* Value
Unadjusted	1.217	0.786–1.646	<0.0001 *	1.492	0.994–1.989	<0.0001 *	1.968	1.318–2.618	<0.0001
Model 1	0.938	0.500–1.376	<0.0001 *	1.221	0.715–1.726	<0.0001 *	1.679	1.013–2.344	<0.0001
Model 2-a	0.968	0.529–1.406	<0.0001 *	1.195	0.689–1.702	<0.0001 *	1.637	0.968–2.304	<0.0001
Model 2-b	0.563	0.070–1.057	0.025 *	0.832	0.261–1.402	0.004 *	1.260	0.538–1.981	0.001

Model 1: adjusted for age, gender, and BMI; Model 2-a: adjusted for Model 1 + AHI_REM_; Model 2-b: adjusted for Model 1 + AHI_NREM_. Diagnostic criteria: REM-related OSA 1: overall AHI ≥ 5 and AHI_REM_/AHI_NREM_ ≥ 2; REM-related OSA 2: overall AHI ≥ 5, AHI_REM_/AHI_NREM_ ≥ 2, and AHI_NREM_ < 15; and REM-related OSA 3: overall AHI ≥ 5, AHI_REM_/AHI_NREM_ ≥ 2, AHI_NREM_ < 8, and REM sleep duration > 10.5 min. Abbreviations: CI, confidence interval. * *p* < 0.05.

**Table 3:** Association between three different definitions of REM-related OS) and the Pittsburgh Sleep Quality Index score in male patients.

	REM-Related OSA 1			REM-Related OSA 2			REM-Related OSA 3		
	Coefficient	95% CI	*p* Value	Coefficient	95% CI	*p* Value	Coefficient	95% CI	*p* Value
Unadjusted	0.500	−1.054–1.027	0.063	0.684	0.061–1.307	0.032	1.057	0.183–1.932	0.018
Model 1	0.426	−0.103–0.955	0.114	0.637	0.013–1.262	0.045	1.048	0.168–1.927	0.020
Model 2-a	0.463	−0.066–0.992	0.086	0.592	−0.033–1.216	0.063	0.970	0.090–1.851	0.031
Model 2-b	−0.030	−0.624–0.564	0.922	0.152	−0.547–0.850	0.670	0.424	−0.516–1.363	0.376

Model 1: adjusted for age and BMI; Model 2-a: adjusted for Model 1 + AHI_REM_; Model 2-b: adjusted for Model 1 + AHI_NREM_. Diagnostic criteria: REM-related OSA 1: overall AHI ≥ 5 and AHI_REM_/AHI_NREM_ ≥ 2; REM-related OSA 2: overall AHI ≥ 5, AHI_REM_/AHI_NREM_ ≥ 2, and AHI_NREM_ < 15; and REM-related OSA 3: overall AHI ≥ 5, AHI_REM_/AHI_NREM_ ≥ 2, AHI_NREM_ < 8, and REM sleep duration > 10.5 min.

**Table 4:** Association between three different definitions of REM-related OSA and the Pittsburgh Sleep Quality Index score in female patients.

	REM-Related OSA 1			REM-Related OSA 2			REM-Related OSA 3		
	Coefficient	95% CI	*p* Value	Coefficient	95% CI	*p* Value	Coefficient	95% CI	*p* Value
Unadjusted	1.828	1.039–2.617	<0.0001	2.155	1.279–3.031	<0.0001	2.436	1.393–3.480	<0.0001
Model 1	1.775	0.985–2.565	<0.0001	2.113	1.233–2.992	<0.0001	2.382	1.333–3.432	<0.0001
Model 2-a	1.778	0.984–2.572	<0.0001	2.126	1.244–3.008	<0.0001	2.410	1.353–3.468	<0.0001
Model 2-b	1.548	0.646–2.450	0.001	1.930	0.906–2.954	0.001	2.185	0.992–3.378	0.001

Model 1: adjusted for age and BMI; Model 2-a: adjusted for Model 1 + AHI_REM_; Model 2-b: adjusted for Model 1 + AHI_NREM_. Diagnostic criteria: REM-related OSA 1: overall AHI ≥ 5 and AHI_REM_/AHI_NREM_ ≥ 2; REM-related OSA 2: overall AHI ≥ 5, AHI_REM_/AHI_NREM_ ≥ 2, and AHI_NREM_ < 15; and REM-related OSA 3: overall AHI ≥ 5, AHI_REM_/AHI_NREM_ ≥ 2, AHI_NREM_ < 8, and REM sleep duration > 10.5 min.
